# Scalp cooling success in a Black woman

**DOI:** 10.1016/j.jdcr.2024.05.008

**Published:** 2024-05-16

**Authors:** Michaela Crawford, Tiffany Mayo

**Affiliations:** a School of Medicine, Meharry Medical College, Nashville, Tennessee; b Department of Dermatology, University of Alabama at Birmingham, Birmingham, Alabama

**Keywords:** alopecia, Black, chemotherapy-induced alopecia, hair loss, scalp cooling

## Introduction

Chemotherapy-induced alopecia (CIA) is emotionally distressing and plays an important role in one’s body image, emotional state, and quality of life.^1^ Forty-seven percent of women consider CIA the most taxing part of receiving chemotherapy, and 8% would forgo chemotherapy due to the fear of alopecia.^2^ Scalp cooling (SC) is a process where a tight-fitted cap is worn on the head during chemotherapy treatments and cooled to a low temperature. It has been shown to decrease CIA through vasoconstriction, reducing both blood perfusion and the amount of chemotherapy absorbed into follicular cells.^3^ Also, the cold temperatures decrease cellular metabolic activity, diminishing the cytotoxic effects of chemotherapy on follicular cells.^3^ In 2017, scalp cooling devices received US Federal Drug Administration approval after studies demonstrated efficacy in preventing CIA.^4^ This improvement has not been generalizable to Black patients, however. Only a few Black patients have participated in SC trials.^4^ One study performed in Black patients showed a lack of efficacy in SC preventing CIA (more than 50% hair loss), prompting most participants to end SC before chemotherapy completion.^4^ Differences in hair texture, shape, and density among ethnic groups may contribute to the lower efficacy of SC seen in Black patients. Here, we report a case examining the success of SC in a Black woman undergoing chemotherapy for breast cancer.

## Case report

A 65-year-old woman diagnosed with stage I breast cancer underwent 4 cycles of intravenous docetaxel and cyclophosphamide chemotherapy and scalp cooling using a proprietary device. Prior to diagnosis, she had chemically relaxed hair. Upon review of the standard hair preparation protocol for the device, she made minor modifications to the regimen. The recommended shampoo was substituted with a hydrating shampoo formulated for ethnic hair. Between chemotherapy cycles, instead of using the proprietary brush, she maintained her hair in multiple twists and finger-combed her hair. She washed her hair once a week and applied oil to her hair and scalp due to dryness and irritation. The patient did not apply heat to her hair and did not receive chemical relaxers during this time. She underwent 4 rounds of chemotherapy concurrently with SC treatments.

**Fig 1:**
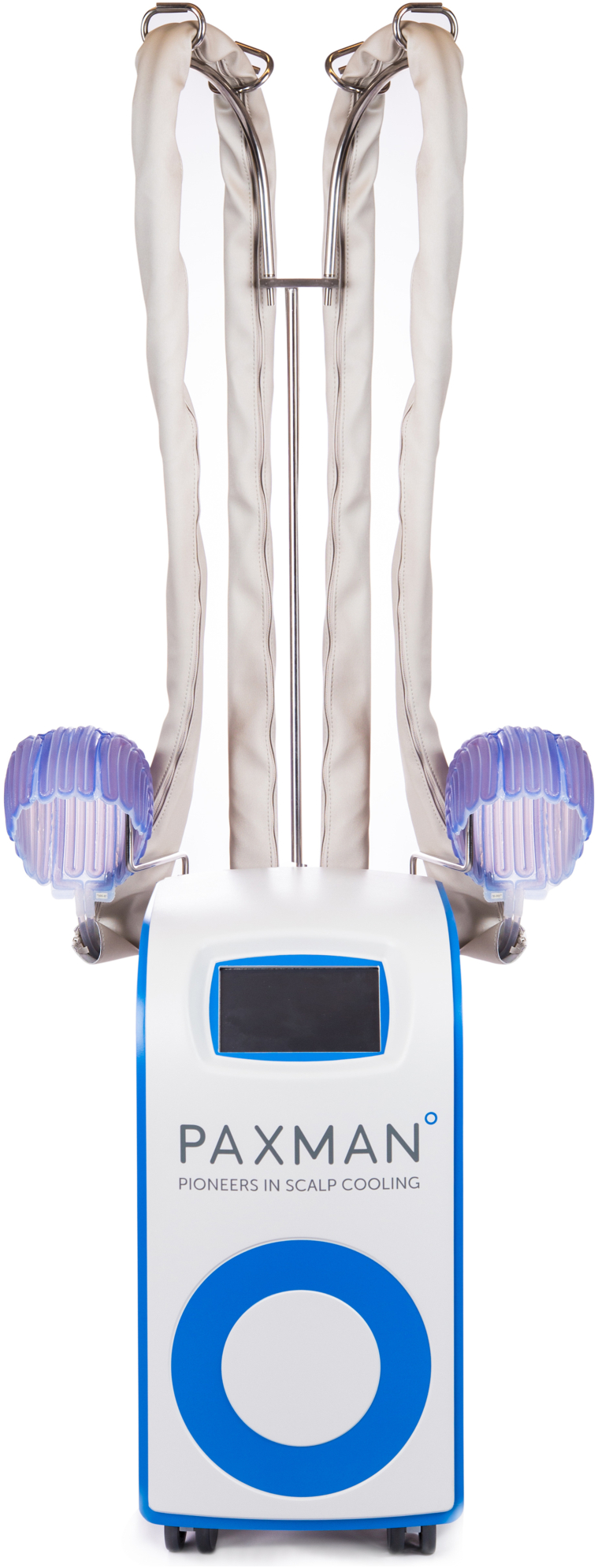
The Paxman Scalp Cooling System. The *blue* inner silicone cap is designed to be in direct contact with and mold to the patient’s head. It contains a network of channels that fill with coolant once connected to the control system. An outer cover is securely fastened over the inner cap.

SC was performed using the Paxman Scalp Cooling System (Fig 1).^5^ An appropriately sized cap was fitted in accordance with the scalp cooling system’s recommendations. The patient wet her hair using the recommended conditioner immediately before placing the cap on her head to decompress the hair, allowing for direct contact between the cap and scalp. SC was initiated 30 minutes before the chemotherapy infusion. The device maintains scalp temperatures at approximately 18 °C by circulating coolant throughout the cap at −4 °C.^5^ Upon chemotherapy infusion completion, scalp cooling continued for 60 minutes. The patient washed her hair 4 to 6 hours after chemotherapy treatment ended, per standard protocol.

**Fig 2:**
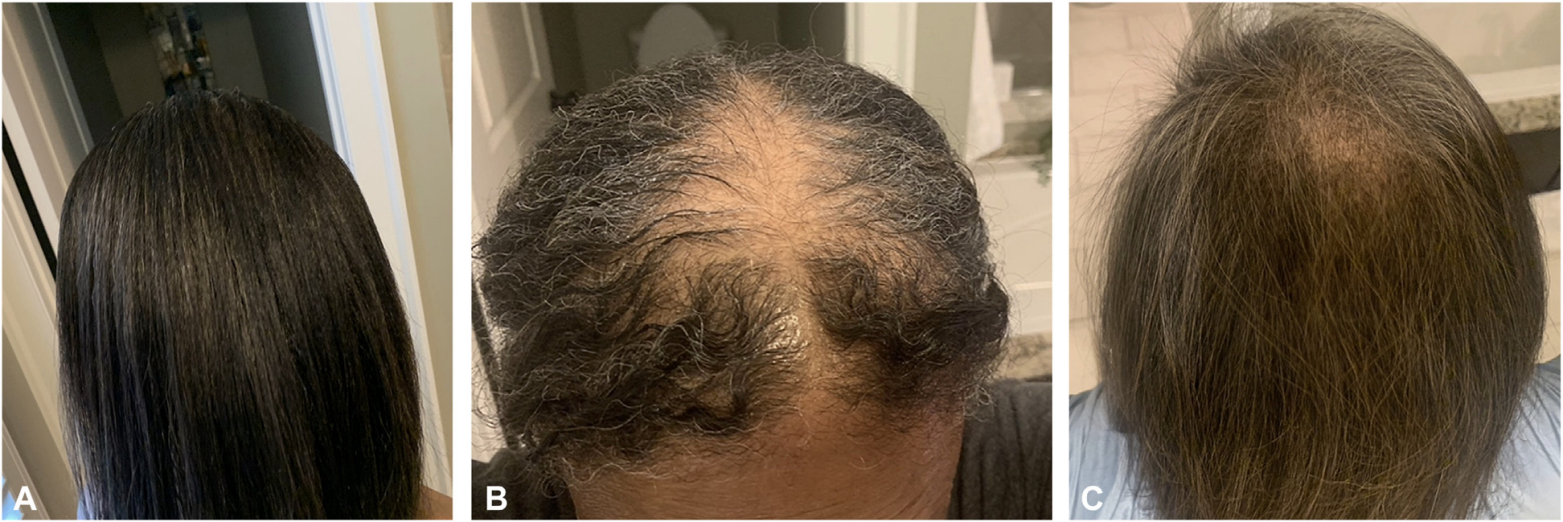
A visual representation of the amount of chemotherapy-induced alopecia (CIA). **A,** Baseline; (**B**) immediately after the last round of chemotherapy; (**C**) 5 months after the last round of chemotherapy.

Given the importance of preventing and/or reducing CIA, successful hair preservation is defined as using SC throughout the duration of all chemotherapy treatments with less than or equal to 50% hair loss.^6^ Treatment failure is defined as the discontinuation of SC due to adverse effects or more than 50% hair loss.^6^ Hair loss (HL) was assessed by both self-evaluation and medical personnel by means of the Dean’s scale^7^: grade 0 (no HL), 1 (<25% HL), 2 (25% to 50% HL), 3 (50% to 75% HL), and 4 (>75% HL) using photographs taken by the patient before chemotherapy and immediately after treatment. A Dean’s scale score of 0 to 2 (HL ≤ 50%) was considered a success.^7^ The patient self-reported an estimated 40% hair loss, noting retention of most of her hair. Medical personnel estimated a Dean’s scale score of 2 (25% to 50% hair loss). Our patient used SC throughout her treatments, was satisfied with her results overall, and experienced less than 50% hair loss, attaining the criteria for success (Fig 2).

## Discussion

Only 12% of the 182 patients enrolled in the Scalp Cooling Alopecia Prevention trial identified as Black.^8^ Another study performed in Dutch hospitals enrolled 1411 patients, but only 1% of patients had “African” hair.^9^ Neither of these studies offered results by race. Only one study was performed on explicitly Black patients to address this issue. The Paxman Scalp Cooling trial examined the efficacy of SC in 15 Black patients. The results showed a lack of efficacy in preventing CIA due to grade 3 alopecia (more than 50% hair loss), prompting most participants to end SC before chemotherapy completion, and only one participant was successful in preventing significant hair loss.^4^

The efficacy of SC largely depends on the patient’s hair type, SC system, proper cap fitting, chemotherapy regimen, dosing, and adherence to directions.^3^ Studies have shown that the type of chemotherapy contributes to either hair preservation or loss. Taxane and nonanthracycline regimens are the most helpful in preventing CIA,^4^ while anthracycline regimens are the least helpful at preventing CIA.^3^

Here, we report a case of a Black female with successful results from SC after 4 rounds of intravenous chemotherapy with docetaxel and cyclophosphamide using a proprietary device, with an estimated hair loss of 40%. Factors that may have contributed to successful SC include a loose curl pattern allowing a tighter fitting cap (due to a prior history of chemical relaxers), taxane-based versus anthracycline chemotherapy regimens, gentle hair care practices, and modifying hair products to include hydrating shampoo and hair oils typically used to groom tightly coiled hair.

Variations in hair texture, volume, shape, and limitations of the cold cap design may correlate to the differences in outcomes when preventing CIA. Straight hair may allow the cooling cap to lay flat on the head for maximum effectiveness. Improvements in the cap design may allow for diverse hair types to achieve greater hair retention. An enriched SC kit with unique products and better-fitting caps explicitly designed for ethnic hair may be a way to combat CIA for more patients. Additional research is needed to improve the efficacy of SC in Black women, which may alleviate the psychosocial effects of CIA in this population.

## Conflicts of interest

Drs Crawford and Mayo received clinical research and grants from Acelyrin, BMS, ChemoCentryx, Galderma, Janssen, Lilly, and Pfizer; received consulting fees from Abbvie, Arcutis, BMS, Bodewell, Janssen, Lilly, Leo Novartis, Pfizer, Physicians’ Education Resource (PER), and UCB; is a board member of the Association of Clinical Research Professionals (ACRP), Southeastern Consortium for Dermatology, and Skin of Color Society; is the chair for the Skin of Color Bylaws Committee; and is an editorial board member of the Journal of the American Academy of Dermatology.
